# Reimagining Post-COVID-19 Continuity of Care for Bipolar Disorder: Nursing Strategies to Address Trauma and Chronic Stress

**DOI:** 10.3390/healthcare13172191

**Published:** 2025-09-02

**Authors:** Maria Karanikola, Maria Nystazaki, Anna Hatzioannou

**Affiliations:** 1 Nursing Department, School of Health Sciences, Cyprus University of Technology, Limassol 3036, Cyprus; 2 General University Hospital Attikon, 124 62 Chaidari, Greece; nystazaki@yahoo.gr; 3 Medical School, University of Nicosia, Nicosia 2417, Cyprus; hatzioannou.a@hotmail.com

**Keywords:** carers, informal caregivers, families, bipolar disorder, chronic stress, nursing, intervention, post-COVID, mental health, continuity of care

## Abstract

According to a recent WHO survey, approximately 93% of countries reported disruptions in mental healthcare provision in the post-COVID-19 era. These have had a significant impact on individuals living with bipolar disorder (BD), many of whom have encountered substantial barriers to accessing mental health services and pharmacological treatment. These factors have been associated with an increased relapse risk, heightened psychosocial stress, and impaired daily functioning. Emerging research underscores the need for interventions that address the chronic stressors experienced by individuals with BD, particularly in the wake of the global trauma induced by the pandemic. In this context, nursing-led interventions play a crucial role, not only in supporting individuals with BD, but also in engaging families and informal caregivers. These interventions emphasize proactive therapeutic engagement, education on relapse signs, and development of adaptive coping strategies. All these contribute to sustained recovery and improved quality of care. This perspective paper explored the challenges and opportunities in delivering mental healthcare to individuals with BD in the post-pandemic era and outlined targeted, nursing-driven approaches that respond to the long-term mental health burden of trauma and chronic stress.

## 1. Introduction: A Widening Mental Health Gap in the Wake of COVID-19

Over the past two years, the COVID-19 pandemic has significantly disrupted mental healthcare systems worldwide, with individuals facing severe neuro-biological disorders, such as bipolar disorder (BD), bearing a disproportionate share of the burden [1].

Despite concerted efforts by governments to ensure continuity of care, the World Health Organization showed that approximately 93% of the countries they surveyed reported pandemic-related interruptions in mental healthcare services. These disruptions increased psychological vulnerability and created barriers in accessing both psychosocial and pharmacological therapies for individuals with BD, thus leaving many of them more susceptible to a relapse. Notably, nearly one out of three individuals with BD reported serious difficulties in accessing mental healthcare, while approximately one out of five encountered significant obstacles to obtaining pharmacotherapy [2].

Thus, not surprisingly, people with BD experienced notable rates of relapse and care disruption during the pandemic [3]. Specifically, in a one-year prospective cohort of individuals diagnosed with BD, 27% of them had a new mood episode, with manic or hypomanic symptoms (21%) being far more common than depressive ones (6%) [4]. Early in the pandemic, studies of smaller outpatient samples suggested the occurrence of a relapse in about 1 out of 10 patients within a few months, often linked to medication discontinuation [5]. Moreover, access to treatment was sharply curtailed during the pandemic, since although nearly three-quarters of patients expressed interest in telepsychiatry only 1% actually received it, while over 90% of them reported no proactive contact from their providers [4].

Based on the above, this perspective paper contends that the pandemic has magnified chronic stress and trauma in this population and their informal caregivers, as well as longstanding challenges in mental healthcare systems worldwide, particularly concerning the provision of psychosocial support and continuity of care. It argues for a recalibration of healthcare strategies and related policies while underscoring the role of mental health nurses in explicitly addressing chronic stress and promoting resilience in individuals with BD and their families.

### 1.1. Chronic Stress and Fragmented Care: An Enduring Crisis

The pandemic has aggravated existing chronic stress and trauma, already prevalent among people with BD, placing them at a heightened risk for a relapse, functional impairment, and emotional distress [6,7]. Prolonged isolation, disrupted routines, and weakened therapeutic alliances during the pandemic exacerbated the already existing emotional and behavioral instability in this clinical population. The resulting psychosocial burden has been compounded by limited healthcare access, diminished treatment adherence, and the discontinuity of therapeutic relationships, which have all been well-documented by recent research [7,8].

In light of these challenges, there is a pressing need to reframe care strategies for BD management through interventions that explicitly address chronic stress and promote resilience. Thus, mental healthcare services need to shift from crisis containment to trauma-informed, resilience-oriented interventions. Trauma-informed care is a clinical framework that recognizes the pervasive impact of trauma on individuals’ mental health and development; this approach is focused on how traumatic experiences determine and shape psychological functioning across the lifespan [9]. Within clinical practice, it seeks to create environments that prioritize safety, empowerment, and collaboration, thereby promoting recovery while minimizing the risk of re-traumatization [9]. Mental health nursing, grounded in holistic and person-centered care, is well-positioned to lead mental healthcare systems’ transformation within this context [10].

### 1.2. Nurse-Led Interventions: Centering Engagement and Emotional Recovery

Mental health nursing is ideally suited to implementing care approaches that respond to chronic stress. Nursing-led interventions that engage both patients and their families are expected to play a central role in enhancing functioning, reducing the relapse risk, and promoting recovery in individuals with BD [11]. These interventions include proactive patient engagement, education on early warning signs of relapse, and equipping individuals with adaptive coping strategies, all in line with international post-COVID-19 guidelines for BD management [12]. Importantly, these interventions should also acknowledge and respond to the emotional and psychological toll of long-term stressors intensified by the pandemic, offering support to both patients and their support networks. Indeed, a quasi-experimental study in 120 individuals diagnosed with BD explored the effectiveness of a nurse-led educational intervention including the development of self-care skills, behavioral management strategies, medication adherence plans, and problem-solving skills [13]. The findings of this study supported the existence of significant improvements in psychological well-being and functional abilities in the experimental group [13]. Similarly, a previous experimental study in individuals with BD and their family members also supported the effectiveness of a nurse-led, family-focused intervention in improving patients’ functionality and burden alleviation [11]. In this study, 149 inpatients were interviewed along with their family members. The control group received routine treatment, while the experimental group received routine treatment along with the family-focused intervention for seven sessions, while posttest measurements were conducted at discharge and at one-month and two-month follow-ups.

### 1.3. Digital Innovation as a Therapeutic Bridge

Prior to the pandemic, structured and web-based educational programs had already proven effective in improving quality of life (QOL) and emotional regulation in individuals with BD, thereby promoting their psychological well-being [10,14]. With the rapid advancement in e-health technologies during the pandemic, these digital tools have become more vital than ever for providing accessible, flexible, cost-effective, and scalable mental health support [15].

Web-based platforms, ranging from virtual education to telehealth therapy and self-management applications, offer a promising means of maintaining therapeutic continuity, while also reducing trauma-related stress caused by service interruptions [16]. Web-based interventions may be targeted at individuals with BD and their families alike, ensuring accessibility even in resource-limited settings.

Nursing professionals are critical in facilitating web-based interventions, which could be delivered both in groups and individually [17,18]. From moderating online support groups to delivering remote education and managing digital care plans, nurses’ role in deploying e-health solutions must be expanded and supported institutionally [17,18].

However, further research is needed to better understand the educational needs of individuals with BD and their families and to evaluate the effects of digital interventions on long-term psychosocial functioning. For instance, studies on the effectiveness of web-based interventions and educational programs regarding improvements in QOL and emotional regulation in the family members of individuals with BD are expected to provide valuable data [14].

### 1.4. Limitations in Implementing Digital e-Health Interventions

While nursing-led digital interventions offer promising avenues for improving care regarding long-term stress and trauma in individuals with BD in the post-pandemic context, it is important to recognize their potential limitations. Specifically, not all individuals with BD may have equal access to digital health solutions, especially older and socially deprived individuals, due to factors such as limited digital literacy, socioeconomic disadvantages, or geographic isolation [19]. Although digital tools are often promoted as a solution to geographical isolation by bringing services to remote or underserved areas, in practice, geographic isolation can still be a barrier if it coincides with (a) poor internet infrastructure, for instance, in rural areas with limited broadband access, (b) limited access to digital devices and technical and social support, especially in low-resource settings/communities, and (c) a lack of community centers/mental health centers, libraries, or inpatient/outpatient clinics that could promote digital literacy or provide connectivity. Thus, while digital tools aim to overcome geographic barriers, in some cases they can unintentionally reproduce or exacerbate them if these basic infrastructure needs are not met [20]. Nevertheless, addressing these disparities is essential to ensure that such interventions are equitable and inclusive.

### 1.5. Family Systems Under Stress: An Overlooked Care Priority

The pandemic further augmented the burden on informal carers of individuals facing mental health problems including BD, often family members, due to the reduced availability of formal support services, further intensifying the exposure to prolonged stress conditions for them [21]. These caregivers have to face their own chronic stress and burnout, which is closely interlinked with the psychosocial functioning of individuals with BD and subsequently their clinical outcomes; disruptions in one domain often echo in the other. Importantly, symptom remission in individuals with BD does not always equate to full psychosocial recovery and rehabilitation for either patients or their families [22].

Especially during the pandemic, caregivers of people with BD reported an increased burden and disrupted support. Specifically, a study in India among caregivers of individuals with BD showed high scores regarding their self-assessed burden and self-perceived stress in the middle of the pandemic [23]. Similarly, a national Norwegian survey conducted during the first wave of the pandemic among family members of people with psychosis and BD showed that approximately 81% of the participants reported reduced healthcare access for their family member facing mental health problems, including access to community care. Regarding the information received from healthcare providers about their relatives’ treatment during the lockdown period, almost half of the participants reported that the information was not satisfactory [21].

Nevertheless, family involvement in BD management is crucial. Thus, supporting family members is vital for alleviating their burden as well as for securing an optimal clinical course for patients. The evidence on the effectiveness of family-targeted interventions in achieving this dual goal is strong. Specifically, a recent review of 47 studies documented that education of both patients and family members was linked with reduced numbers of new mood episodes, as well as diminished numbers and durations of hospitalizations, also associated with the experienced burden [24].

Thus, we propose that post-pandemic mental health services need to prioritize not only the individual but also the family system. This involves the active integration of families into care planning, providing them with comprehensive support that includes BD-specific education and the development of stress management competencies, further supporting their mental well-being alongside that of their loved ones.

### 1.6. Sustaining Pharmacological Adherence Through Nurse-Led Programs

Pharmacotherapy remains a foundational component of BD treatment. Lithium continues to be the most evidence-based mood stabilizer, associated with a reduced relapse risk and enhanced functioning, as well as improved QOL, in both BD-I and BD-II populations [25,26]. Thus, lithium treatment needs to be considered early in the course of the disease [26]. However, pandemic-related barriers and discontinuity of care have made medication adherence more difficult, particularly among underserved populations, such as those facing mental health difficulties.

Nurse-led medication management programs delivered through dedicated clinics, such as lithium or clozapine clinics, telehealth follow-ups, and family-centered pharmacotherapy education programs, can address this gap. The above initiatives improve understanding of treatment regimens, promote shared responsibility and co-management of treatment plans, and enhance patients’ adherence to therapy [27]. For instance, a single-blind, randomized controlled trial by Balikai et al. [28] supported the positive impact of a nurse-led intervention on promoting medication adherence behavior and a subsequent reduction in symptom severity among 85 inpatients with BD. Specifically, this study provided evidence for improved medication adherence behavior, followed by a significant decrease in symptom severity, in the participants due to the nurse-led intervention, further resulting in better clinical outcomes, prevention of relapses, and decreased numbers of readmissions. Indeed, according to a relevant review, numerous studies document that nurse-led educational interventions in patients with BD are associated with improved adherence to drug treatment and optimal clinical outcomes [24].

As cost-effective and accessible models, nurse-led educational interventions can be embedded into broader strategies for ensuring continuity of care. Overall, nurse-led medication management programs may be deemed as essential to ensuring continuity of care in resource-strained systems.

## 2. Conclusions: Nurses as Catalysts for Post-Pandemic Reform

As we transition beyond the acute phases of the pandemic, the enduring psychosocial stress in individuals with BD and their families remains a significant concern. Chronic stress, exacerbated by isolation, uncertainty, emotional trauma, and service gaps, demands immediate attention and coordinated responses.

This perspective paper argues that nurses must lead initiatives to build trauma-informed, digitally empowered, family-inclusive systems of care. Their clinical expertise, therapeutic engagement, and proximity to both patients and their families uniquely position them as catalysts for structural reform in mental healthcare that centers around resilience-building, digital innovation, and collaborative care for those diagnosed with BD and their families.

To fully realize this potential, support must be provided across multiple levels [29]. At the system level, healthcare policies must allocate resources for advanced nursing education in mental health and family care and integrate nurses into decision-making processes around service design and delivery. At the organizational level, institutions should foster interdisciplinary collaboration, ensure manageable caseloads, and embed nurse-led models aligned with integrated and family-centered care principles within routine community mental health services. At the interpersonal level, nurses need to be supported to facilitate proactive engagement with individuals and families, by providing education, identifying early warning signs, and cultivating resilience through person-centered care.

Redesigning mental healthcare services to be inclusive, resilient, relationship-centered, and rooted in recovery is not only a clinical necessity but also an ethical imperative. By investing in multi-level provisions for nursing-led interventions that foreground psychosocial recovery and family involvement, post-COVID-19 mental healthcare systems can become more humane, equitable, and future-ready.

Nevertheless, while digital and nursing-led interventions have clear promise in addressing long-term stress and trauma in individuals with BD, it is important to acknowledge their potential limitations. Addressing relevant challenges and gaps is essential in ensuring equitable and inclusive mental healthcare in the post-pandemic era.

## Data Availability

No new data were created or analyzed in this study.
